# Exploring Marine Natural Compounds: Innovative Therapeutic Candidates Against Chagas Disease Through Virtual Screening and Molecular Dynamics

**DOI:** 10.3390/life15020192

**Published:** 2025-01-28

**Authors:** Carlos Eliel Maya-Ramírez, Asmae Saih, Alfonso Méndez Tenorio, Carlos Wong Baeza, Benjamín Nogueda Torres, Juan Carlos Santiago Hernández

**Affiliations:** 1 Laboratorio de Diagnóstico Molecular, Departamento de Bioquímica, Escuela Nacional de Ciencias Biológicas, Instituto Politécnico Nacionals, Mexico City 11340, Mexico; emayar1800@alumno.ipn.mx; 2 Laboratory of Biology and Health, URAC 34, Faculty of Sciences Ben M’Sik, Hassan II University of Casablanca, Casablanca 20250, Morocco; asmae.saih-etu@etu.univh2c.ma; 3 Laboratorio de Biotecnología y Bioinformática Genómica, Departamento de Bioquímica, Escuela Nacional de Ciencias Biológicas, Instituto Politécnico Nacional, Mexico City 11340, Mexico; amendezt@ipn.mx; 4 Laboratorio de Biomembranas, Departamento de Bioquímica, Escuela Nacional de Ciencias Biológicas, Instituto Politécnico Nacional, Mexico City 11340, Mexico; charlywong@icloud.com; 5 Laboratorio de Helmintología, Departamento de Parasitología, Escuela Nacional de Ciencias Biológicas, Instituto Politécnico Nacional, Mexico City 11340, Mexico

**Keywords:** Chagas disease, *Trypanosoma cruzi*, marine natural compounds, molecular docking, PCA, pharmacokinetics

## Abstract

Chagas disease, caused by the protozoan *Trypanosoma cruzi*, represents a significant public health challenge, particularly in Latin America’s endemic regions. The limited efficacy and frequent adverse effects of current treatments underscore the need for novel therapeutic options. This research explores marine natural compounds as potential candidates for Chagas disease treatment using virtual screening and in silico evaluation methods. Techniques such as molecular docking, drug-likeness evaluation, and pharmacokinetic analysis were employed to identify promising anti-parasitic compounds. Among the candidates, chandrananimycin A, venezueline A, and dispacamide demonstrated high binding affinities to key targets in *T. cruzi* alongside favorable docking scores and compliance with essential drug-likeness criteria. Pharmacokinetic profiling further supported their therapeutic potential, revealing desirable properties like effective absorption and minimal toxicity. These findings underscore the promise of marine-derived compounds as a valuable source of new drugs, emphasizing the need for further in vitro and in vivo investigations to elucidate their molecular mechanisms and optimize their development as viable treatments for Chagas disease.

## 1. Introduction

The protozoan *Trypanosoma cruzi* (*T. cruzi*) induces Chagas disease (CD), a significant health challenge in Latin America that affects 6–8 million individuals and puts another 75 million at risk of infection [1]. The disease predominantly occurs in endemic regions across 21 Latin American countries, such as Mexico, Argentina, Brazil, Bolivia, and Chile; however, global warming and migration facilitate its propagation across different worldwide areas [2]. The World Health Organization (WHO) has designated CD as a Neglected Tropical Disease, emphasizing the critical need for enhanced priority, focus, and funding [3]. About 30% of infected individuals could develop gastrointestinal and/or heart issues. Notably, just 1% of *T. cruzi* carriers receive medical treatment, and only 3% of carriers are detected [4].

The protozoan *Trypanosoma cruzi* has four separate stages in its life cycle. These are the epimastigote, the metacyclic trypomastigote, the amastigote, and the bloodstream trypomastigote. This cycle occurs within both vertebrate and invertebrate hosts (the mammalian and the triatomine hosts), and categorized into replicative or infective phases [5].

CD has two stages: the acute phase, which typically takes 4–8 weeks and is frequently mild or asymptomatic, and the chronic phase may manifest 10 to 20 years post-initial infection [6]. The first phase remains undetected, and 95% of individuals infected with this parasite are typically asymptomatic [7]. Only general symptoms are evident, such as fever, diarrhea, headache, nausea, vomiting, and cephalalgia. The last phase is characterized by low parasitemia, with diagnosis dependent on the patients’ immune response [8].

Over many years, approximately 30 to 40% of infected individuals exhibit cardiac and/or digestive disorders that may result in mortality. Some molecular and cellular processes that have not been fully figured out yet are to blame for these disorders. These include the parasite’s ability to stay in the host, an imbalance in the host’s immune system, and genetic effects on both the parasite and the host [9].

Currently, most countries routinely use two pharmacological therapies for CD treatment: benznidazole (BZN) and nifurtimox (NFX), though their availability may vary depending on national regulatory approvals [10]. The treatment protocol using BZN or NFX is protracted and associated with several adverse effects, resulting in issues with patient adherence [11]. During the acute phase of infection, both BZN and NFX demonstrate efficacy. Still, their benefits diminish during the chronic phase due to tropism, immunological variability, drug resistance, parasite genetic diversity, and efficacy variation [12,13]. This underscores the urgent need for the development of novel, affordable, and effective drugs.

Marine natural products (MNPs) derive from different marine organisms: microorganisms, marine animals, and plants. Mostly used are alkaloids, steroids, terpenoids, lactones, peptides, and polyketones which are from marine organisms. Such MNPs have been found out as possible sources of novel bioactive agents showing potential properties, such as antiparasitic, antiviral, antifungal, antileishmanial, antiplasmodial, and antitrypanosomal for potential therapeutic applications [14].

Molecular docking has become a fundamental component of in silico drug discovery. This computational approach involves predicting the interactions between a ligand and a receptor at the atomic level. The docking process consists of two primary steps: the search algorithm and the scoring function. The urgent need to develop new treatments for CD has driven researchers to explore unconventional sources of bioactive compounds, with the marine environment emerging as a particularly promising reservoir. This study is based on the hypothesis that chemical compounds derived from marine organisms, identified through bioinformatics tools such as virtual screening and molecular docking, can act as effective and selective inhibitors of the TcBDF2 bromodomain of *Trypanosoma cruzi*. This bromodomain plays a critical role in the parasite’s epigenetic regulation, making it a compelling therapeutic target for drug design.

The primary objective of this research is to explore the chemical richness of the Comprehensive Marine Natural Products Database (CMNPD) to identify potential TcBDF2 inhibitors that could serve as starting points for the development of new therapies. To achieve this, virtual screening techniques were employed to select compounds with high affinities for the bromodomain, followed by molecular docking studies to evaluate key interactions between the selected compounds and TcBDF2. This bioinformatics-driven approach aims to identify lead compounds with therapeutic potential, considering both their specificity and safety profiles, thus contributing to the advancement of treatment strategies for this disease, which affects millions of people in endemic regions, which are represented as a graphical abstract.

## 2. Materials and Methods

### 2.1. Data Set

Marine compounds were downloaded from the CMNPD (www.cmnpd.org, accessed on 1 October 2024) in the Structure Data File (SDF) format. The CMNPD serves as a manually curated, open-access knowledge base for research on marine natural products. This database provides a large number of compounds, each with unique structures and characteristics, making them suitable candidates for virtual screening and computational analysis. Marine compounds demonstrate various pharmacological activities, including antiparasitic, antibacterial, antidiabetic, antifungal, and anti-inflammatory effects, making them an important starting point for the identification of new treatments [15].

### 2.2. Ligand Preparation

Ligand preparation is a critical and essential step in molecular docking research as it ensures that the resulting structures are highly quality and suitable for use in drug design studies [16]. In this study, the 3D structure of the compounds downloaded from the CMNPD were optimized using Avogadro 4.0 [17], with parameters set to run the steepest descent algorithm and energy minimization was achieved using the MMFF94 force field. The protonation state was set to 7.4, to ensure that the molecules were in a physiologically relevant state.

### 2.3. Receptor Preparation and Identification of the Active Site

The X-ray crystallography structure of *T. cruzi* TcBDF2 (PDB code: 6NIM; Resolution: 1.78 Å) was downloaded from the RSCB Protein Data Bank (https://www.rcsb.org/, accessed on 5 October 2024) in PDB format [18]. In selecting this specific structure, we paid attention to the need for the most robust and well-validated crystal structure, thereby ensuring the accuracy and reliability of subsequent analyses. Chimera 1.16 software [19] (https://www.cgl.ucsf.edu/chimera/) was used to prepare the downloaded protein for molecular docking. Protein preparation steps included the removal of water molecules, metal ions, and other artifacts so as to simplify docking calculations. Charges and hydrogen atoms were assigned to the protein residues, using default settings. In addition, any residues with structural anomalies were rectified. Energy minimization was then performed to optimize the structure, using the steepest descent and conjugate gradient steps, and the final structure was exported in PDB format for molecular docking studies. The binding site of the prepared protein was determined using the AutoSite tool 1.0 [20] (https://ccsb.scripps.edu/autosite/).

### 2.4. Molecular Docking Process

Molecular docking (virtual screening) was performed using Autodock Vina 1.2.3 [21] from the PyRx 0.8 suite [22]. Briefly, the PDB file of the prepared target protein was loaded in PyRx 0.8 and then converted into a macromolecule in the PDBQT file format. The ligand structures in SDF file format were subjected to energy minimization before being converted to the PDBQT format using the OpenBabel 2.4.1 [23] within PyRx 0.8. Molecular docking was executed within the Vina workspace in a grid box with the size of 20 Å x 20 Å x 25 Å fixed on the active site with coordinates (x, y, z) 6.153, 74.347, 15.781. The exhaustiveness was set to the default at 16 [24]. The final results were analyzed, focusing on the ligands that showed the best affinity scores and the most promising interactions with the receptor protein, and visualized using Discovery Studio 21.1.0.20298 [25] and ProteinsPlus (PoseView), accessed on 8 November 2024 [26].

### 2.5. Drug Likeness Analysis and ADMET Predictions

The evaluation of the drug-like and central absorption–distribution–metabolism–excretion–toxicity (ADMET) properties of drug candidates is an essential step in drug discovery and development [27]. To assess these properties, we used pkCSM [28] to determine the drug-likeness properties of 31 high scoring ligands. The Lipinski’s rule of 5 (RO5) [29], which is regarded as a rule of thumb in computer-aided drug discovery, was used to screen the compounds. In addition to Lipinski RO5, we also used the SWISS ADME, accessed on 10 November 2024 server [30] to filter out false positives (PAINS structures) from the screened hits. Compounds with desirable drug-likeness and ADMET properties were subjected to MDs.

### 2.6. Molecular Dynamics Simulations

To perform the 500 ns MDs for the complex with the ligands obtained from CMNPD, the protein’s crustal structure (PDB 6NIM) was downloaded from the Protein Data Bank website. The preparation of the protein and the ligands was performed using CHARMM-GUI [31], and the protein–ligand complex option was selected. The ligand structures were loaded in PDB format, and CHARMM-GUI automatically applied the CHARMM36 force field for each one [32]. The system was neutralized by adding counterions with a concentration of 0.15 M NaCl to simulate physiological conditions [33]. Then, a cubic simulation box was defined with a minimum of 10 Å between the protein surface and the edges of the box. The box was solvated with TIP3P water molecules [34], and the necessary files for GROMACS [35] (protein and ligand topologies, parameter files, and initial settings) were generated through CHARMM-GUI. Energy minimization was carried out using GROMACS to eliminate collisions or initial stresses in the system. Subsequently, an equilibrium stage was carried out in two phases. First, an NVT assembly was applied to gradually heat the system up to 310 K, stabilizing the temperature. Then, an NPT assembly at 310 K and 1 atm was used for pressure equalization. After equilibration, production simulation was carried out for 500 ns in the NPT assembly at 310 K [36,37]. The structural stability of the complex was evaluated by calculating the root mean square deviation (RMSD). At the same time, the flexibility of individual residues was analyzed with the root mean square fluctuation (RMSF). Solvent accessible surface area (SASA), radius of gyration (Rog), and interactions between the TcBDF2 bromodomain and ligands were also examined using hydrogen bonding analysis. The principal component analyses (PCA) and residue cross-correlation analyses (DCCM) were performed using the Bio3D library in the R environment. For the PCA, the covariance matrix of the atomic positions was obtained from molecular dynamics trajectories previously processed in GROMACS. With this matrix, the eigenvalues and eigenvectors were calculated to identify the main modes of movement of the system, which were graphically represented in two dimensions for the first principal components. The cross-correlation analysis (DCCM) was carried out from the same covariance matrix. Pearson correlations were calculated between the displacements of the residues throughout the simulation, obtaining a map that describes the dynamic relationships between different parts of the protein.

## 3. Results and Discussion

### 3.1. Molecular Docking Simulation

Molecular docking, a widely used computational technique in drug discovery, predicts the binding affinity of small molecules (ligands) to receptor proteins [38], offering valuable insights into ligand behavior within a protein’s binding pocket and the biochemical mechanisms of their interaction. In this study, molecular docking simulations were employed to examine the interactions between optimized compounds from the CMNPD database and the TcBDF2 target. Table 1 highlights the top 31 hit molecules, all of which exhibit superior binding affinities compared to the reference ligand, underscoring their potential as promising candidates for further development.

### 3.2. Drug-likeness and In Silico ADMET of Potential Hit Compounds

#### 3.2.1. Drug-likeness

We systematically assessed the ligands in silico drug-likeness and toxicity predictions to determine their suitability for pharmacological applications. Evaluating drug-likeness parameters is an important step in the initial stages of drug discovery, aiming to minimize the loss of time and resources. In computer-aided drug discovery (CADD), in silico screening links the physicochemical properties of a compound to its pharmacological behavior, particularly its oral bioavailability. According to Lipinski’s Rule of Five (RO5), a compound is considered likely to be orally bioavailable if it does not violate more than one of the following criteria: (i) no more than 5 hydrogen bond donors, (ii) no more than 10 hydrogen bond acceptors, (iii) a molecular weight under 500 daltons, and (iv) a calculated octanol–water partition coefficient (Clog P) not exceeding 5 [39]. In addition to the bioavailability assessments, we also screened the shortlisted compounds for pan-assay interference compounds (PAINS) [40]. Compounds classified as PAINS (Pan-assay interference compounds) often exhibit apparent bioavailability but are considered promiscuous due to their ability to engage in multiple, nonspecific interactions. These behaviors can interfere with assay readouts, leading to misleading results and complicating the identification of true pharmacological effects. [41]. All compounds containing PAINS substructures were excluded due to their unsuitability as lead compounds for drug discovery. Of the 30 shortlisted molecules, 26 did not meet the required criteria (Table 1) and were eliminated from further evaluation. Only Compounds 8, 23, 24, 25, and 31 satisfied the drug-likeness parameters established in this work. These five compounds exhibited desirable drug-like properties, as determined by Lipinski’s Rule of Five (RO5), and showed no PAINS alerts. Furthermore, all five achieved a bioavailability score of 0.55, reinforcing their potential as viable candidates for drug development.

#### 3.2.2. ADMET

An optimal drug candidate must be safe for oral administration, exhibit efficient absorption into the bloodstream, and undergo elimination without disrupting normal physiological functions. To ensure that the selected hit molecules are pharmacologically viable and safe for human use, comprehensive in silico ADMET (absorption, distribution, metabolism, excretion, and toxicity) evaluations were conducted. [42,43]. Table 2 presents the ADMET properties obtained through the pkCSM online server. The predicted intestinal absorption values are highly favorable, with all compounds demonstrating absorption rates surpassing the 30% threshold. To assess the permeability of the potential hit compounds through the blood–brain barrier (BBB), the BBB penetration ratings were also considered. A log BB value that is >0.3 indicates simple BBB permeability, while a log BB value < −1 suggests poor BBB distribution. Other than Compound 4, all the compounds had log BB values < 3, suggesting that most of the compounds have relatively poor distribution in the BBB. The CYP450 enzyme superfamily is crucial for the bioactivation and detoxification of pharmaceutical compounds. Inhibition of CYP enzymes can reduce drug metabolism within the body, increasing the risk of toxicity. Among these enzymes, CYP3A4 and CYP2D6 are particularly significant, as they are the primary contributors to phase I drug metabolism in humans.

Regardless of the disease’s origin, our study’s ADME profiling focuses on evaluating the drug-like properties of compounds. In the context of Chagas disease, ADME parameters are critical for assessing the potential efficacy and safety of therapeutic agents targeting *T. cruzi*. For example, adequate drug absorption ensures the drug’s sufficient systemic availability, while favorable distribution helps the compound reach infection sites such as cardiac and gastrointestinal tissues. Metabolic stability is crucial for maintaining therapeutic levels over time, and safe excretion minimizes toxicity. While Chagas disease and other conditions may differ in origin, ADME remains universally relevant as a framework for evaluating pharmacokinetic suitability in drug development [44].

The ADME parameters of Molecules 8 and 23 were evaluated and compared with those of BZN and NFX, the drugs currently used to treat Chagas disease. While these reference compounds have known efficacy, they act on different therapeutic targets (nitroreductases and the kinetoplast) and present limitations in terms of toxicity and specificity. In contrast, Molecules 8 and 23 are specifically designed to target the TcBDF2 bromodomain in *Trypanosoma cruzi*, offering a significant therapeutic advantage. In terms of intestinal absorption, Molecule 8 demonstrated the highest percentage (88.16%), followed by Molecule 23 (80.91%), outperforming BZN (75.5%) and NFX (83.91%). This high absorption level suggests that both molecules have a more favorable pharmacokinetic profile for oral administration, a critical factor for the treatment of chronic diseases such as Chagas disease.

Regarding blood–brain barrier (BBB) permeability, Molecule 8 stood out with a log BB value of −0.141, indicating a higher ability to cross this barrier compared to BZN (−0.863) and NFX (−0.953). This finding is particularly relevant for neurological forms of the disease. In contrast, Molecule 23 exhibited lower permeability (−1.055), which may limit its action in the central nervous system but does not necessarily affect its effectiveness in other tissues. None of the evaluated molecules, including the reference compounds, showed inhibition of the metabolic enzymes CYP2D6 or CYP3A4, significantly reducing the risk of drug–drug interactions. This aspect is particularly important in clinical scenarios where patients may be receiving multiple therapies concurrently. In terms of total clearance, Molecule 23 exhibited the lowest value (−0.067), suggesting slower elimination and potentially prolonged action. On the other hand, molecule 8 (0.334) showed an intermediate clearance rate compared to BZN (0.519) and NFX (0.718), reflecting a potentially optimal balance between action duration and elimination. One of the most significant findings is the absence of AMES toxicity in molecules 8 and 23, in contrast to BZN and NFX, which do exhibit genotoxic potential. This positions molecules 8 and 23 as safer options with a lower risk of genetic toxicity, a critical aspect in the development of new drugs.

Overall, the ADME parameters highlight the importance of Molecules 8 and 23 as promising candidates for the treatment of Chagas disease. Their favorable profiles in absorption, metabolism, and toxicity, combined with their specific therapeutic target (TcBDF2), make them attractive options for further studies, including experimental testing and structural optimization. While both molecules exhibit differences in certain parameters, such as BBB permeability and total clearance, these complementary characteristics could be exploited in future research to develop targeted and safe therapies for this disease.

### 3.3. Interactions Between Potential Hit Molecules and the TcBDF2, Active Site Residues

We made interaction maps to learn more about how the possible hit molecules (Compounds 8, 23, and 31) behave in the binding pocket of TcBDF2. Table 3 presents the residues accountable for the high binding affinities exhibited by the potential hit molecules.

**Figure 1:**
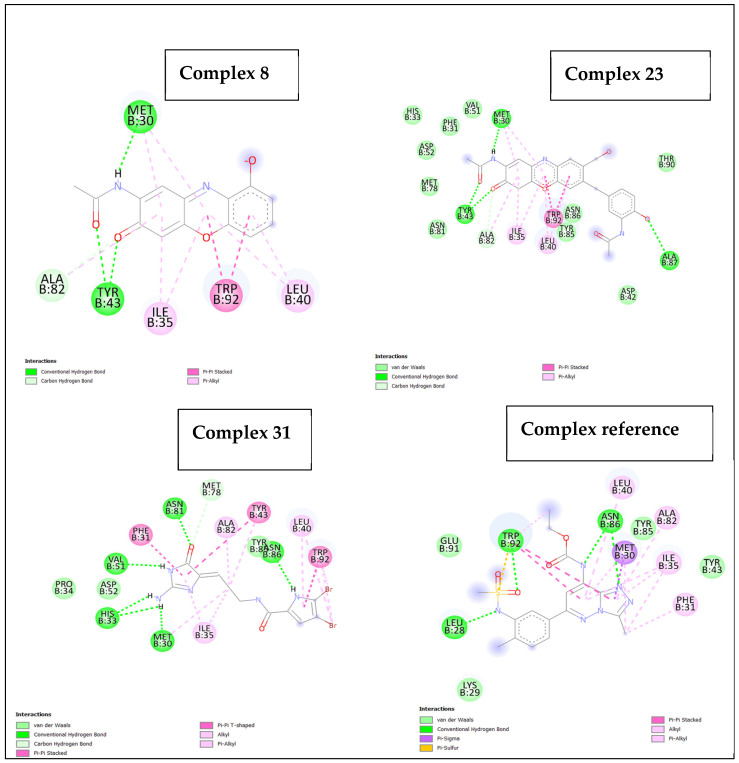
2D interaction map of TcBDF2 bromodomain–ligand complexes. The color code is shown that indicates the type of interaction generated in Discovery Studio, and each complex is also indicated according to the corresponding number in Table 3.

Compound 8 is the most potent ligand (BA = −9.1) among the short-listed compounds, primarily because of its remarkable binding affinities with the TcBDF2 target (Table 3). Notably, we observed that this ligand engaged with the target by forming three conventional hydrogen bonds between the residues Tyr43 and Met30, and two oxygen and a nitrogen atom of the ligand. Hydrogen bonds play an essential role in determining protein-ligand stability and selectivity. The strong binding affinity of Compound 8 is primarily attributed to its ability to form a significant number of hydrogen bonds. Additionally, the ligand engages in other interactions, including carbon–hydrogen bonds, π–π stacking, and π–alkyl bonds, which collectively enhance its binding strength. A detailed two-dimensional representation of the interactions between Compound 8 and the active site residues of the TcBDF2 target is illustrated in Figure 1.

As presented in Table 3, Compound 23 ranks as the second most potent ligand among the screened compounds, with a binding affinity of −8.8 kcal/mol. Its stability within the binding site is primarily supported by three hydrogen bonds involving Tyr43, a critical residue in the interaction with the TcBDF2 target, and Met30. In addition, the ligand established van der Waals interactions with eight residues and other forms of interactions such as carbon–hydrogen bonds, pi–pi-stacked interactions, and pi–alkyl interactions (Figure 1). Compound 31, the third potent ligand with a BA of −8.6, established hydrogen bonds with Met30, His33 (2 H-bonds), Val51, Asn81, and Asn86. The complex was even more stable because of van der Waals interactions with Tyr85, Pro34, and Asp52, and carbon–hydrogen bonds, pi–pi-stacked interactions, and pi-alkyl interactions. The interaction map for ligand 31. In this study, Bromosporine, which serves as the reference molecule, had a BA of −7.7 and displayed interactions with the active site residues of the target protein. Notably, Bromosporine formed a conventional hydrogen bond formation with three residues, Leu28, Trp098, and Asn86 (2 bonds). In addition, the ligand established four van der Waals interactions with Glu91, Lys29, Tyr85, and Tyr43. The reference ligand had other weaker bonds, notably pi–sigma interaction, pi-sulfur interaction, and pi–alkyl interaction. A visual representation of these interactions between Bromosporine and the active site residues of the target protein is shown in Figure 1.

### 3.4. Molecular Dynamics Simulation (MD)

Compounds 8, 23, and 31 were found to be the best drug candidates in this study because they had high docking scores, good pharmacokinetic properties, and stable protein-ligand interactions that were predicted. Hence, we carried out a 500-ns MD simulation to gain a comprehensive insight into the stability of the docked complexes.

#### 3.4.1. Root Mean Square Deviation (RMSD)

**Figure 2:**
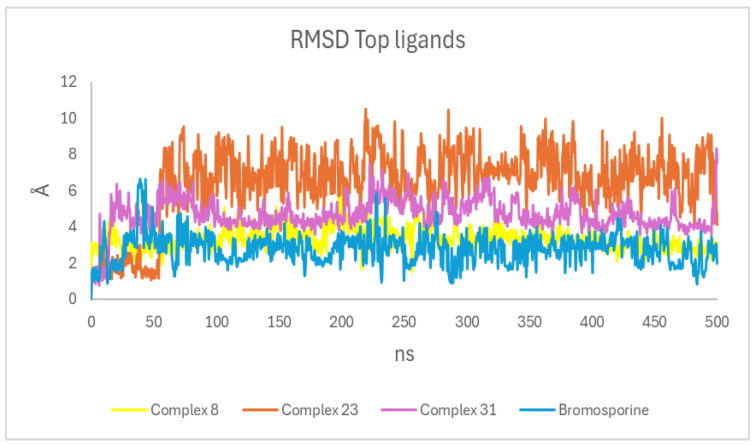
RMSD plot of the ligands, the Y axis in angstroms, and the X axis in nanoseconds indicates the evolution of ligand stability with respect to the protein and its pocket. The plot shows the values of Complexes 8, 23, and 31 and the reference ligand (bromosporine).

During the simulation, we calculated the RMSD to determine the overall stability of the specified systems [45]. In the first 70 ns, the backbone RMSD values for the reference ligand (bromosporine) and the three potential TcBDF2 inhibitors (8, 23, and 31) were between 1 Å and 8 Å. This showed that the molecules changed their shape inside the binding pocket (Figure 2). Consequently, all systems evidently attained the plateau after 30 ns. Then Complexes 23, and 31 showed superior stability when compared to the bromosporine system. This indicates that these two compounds may exhibit greater binding affinity to the TcBDF2 protein than bromosporine.

#### 3.4.2. Root Mean Square Fluctuations (RMSF)

**Figure 3:**
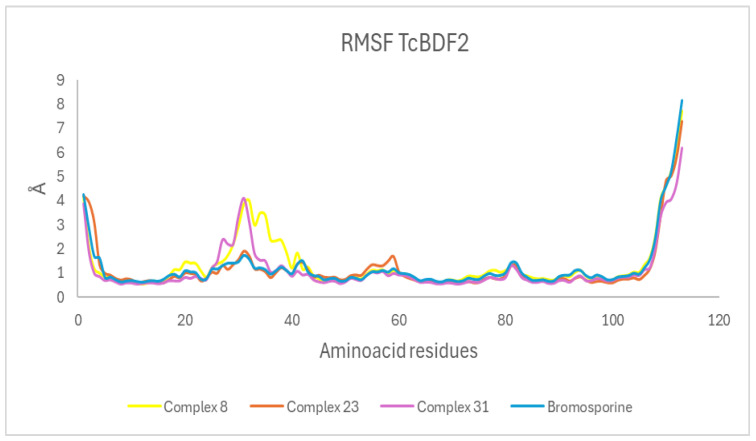
Protein RMSF profiles for the Compound 8, 23, and 31 and bromosporine complexes.

The root mean square fluctuation (RMSF) analyzes protein molecular dynamics trajectories to describe variations in residue flexibility. It calculates the average variation in the locations of individual atoms within a molecule and sheds light on the molecule’s adaptability and dynamic behavior. A higher RMSF value during simulation denotes more flexibility, whereas a lower RMSF signifies more rigidity [46]. Figure 3 illustrates that the RMSF profiles of all systems exhibited considerable similarity. The stability of residues 15–40 and 60–90 showed that these parts were important for the binding of possible inhibitors to TcBDF2 and had stable hydrogen interaction regions. They included significant residues like Met30, His33, Pro34, Tyr43, Met77, Asn81, Tyr85, and Asn86. These residues established H-bonds with the ligands, diminishing the respective domains’ flexibility. We discovered that the three possible TcBDF2 inhibitors had the same

RMSF profiles because they formed hydrogen bonds with Met30 and Tyr43. Our results revealed that the Complexes 8, 23, and 31 exhibited greater stability than the reference TcBDF2 complex (Figure 3).

#### 3.4.3. Radius of Gyration (RoG)

**Figure 4:**
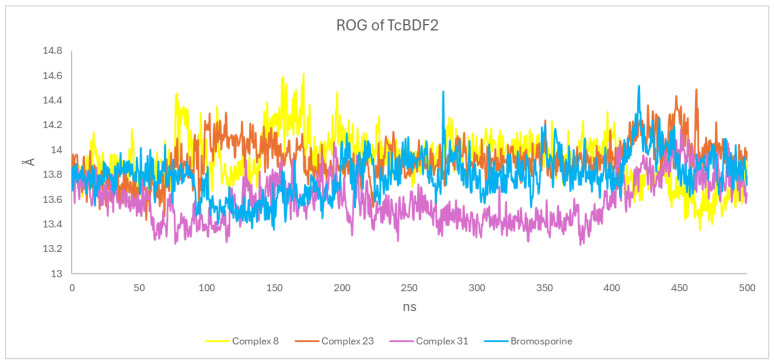
ROG profiles for Compounds 8, 23, and 31 and bromosporine complexes.

We investigated how binding to various compounds changes the protein’s structural compactness. Therefore, we determined the RoG as a function of time. A ligand tends to be flexible and unstable when RoG is high enough. On the other hand, conformations with lower RoG values are typically dense and closely packed [47]. The RoG values for all systems are consistently between 13.2 Å and 14.8 Å, signifying that the TcBDF2 protein retains a compact and stable structure during 500ns. TcBDF2 protein compactness was higher in Compounds 23 and 31 than in bromosporine, which is in line with the findings of RMSD analysis of the protein backbone (Figure 4).

#### 3.4.4. Solvent Accessible Surface Area (SASA)

**Figure 5:**
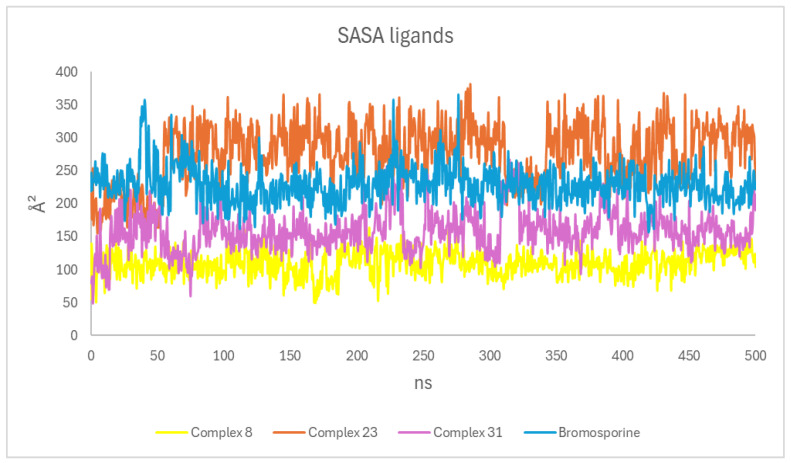
SASA (solvent accessible surface area) profiles for Compounds 8, 23, and 31 and bromosporine complexes.

SASA is a protein property that measures the contact area between the TcBDF2-ligand complexes and the solvent during the 500 ns simulation [48]. The SASA results show that bromosporine (200–300 Å^2^) and Complex 8 (50–150 Å^2^) stay in place and are buried well in the binding pocket, which means they have strong interactions with the TcBDF2 protein. Complex 23 has more solvent exposure (250–400 Å^2^) and more variation, which means it is less stable when it comes to binding (Figure 5).

#### 3.4.5. Hydrogen Bond Analysis

**Figure 6:**
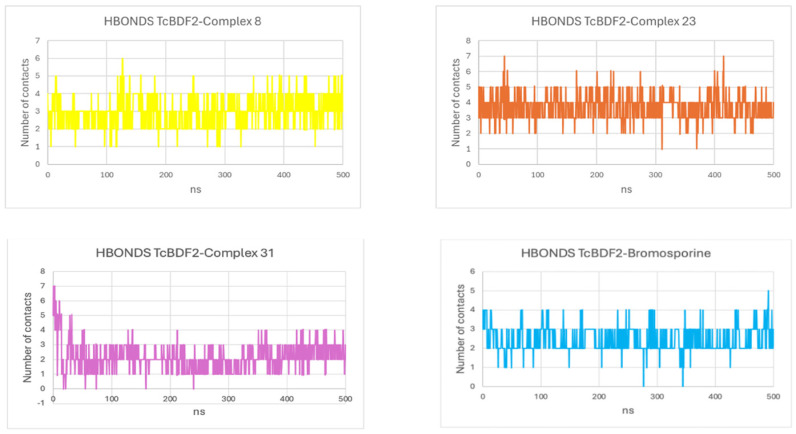
HBONDS (hydrogen bonds) profiles for Compounds 8, 23, and 31 and bromosporine complexes.

The stability of the protein-ligand complex mainly relies on hydrogen bonds, which are highly specific interactions between the receptor and ligand. They are also accountable for drug solubility, metabolism, and adsorption in drug discovery [49]. According to the data, the hydrogen bonds remained strong for all four systems over the simulation run. Additionally, as Figure 6 illustrates, the hydrogen bonds varied from 1 to 7, with averages of 3, 3, 4, and 4, respectively, for Complexes 8, 31, 23, and bromosporine. Compared to the bromosporine, the results showed that the three marine-based compounds that were looked into in this study consistently bind with the target TcBDF2 protein and stay in the binding pocket during the simulation.

#### 3.4.6. Principal Component Analysis (PCA)

**Figure 7:**
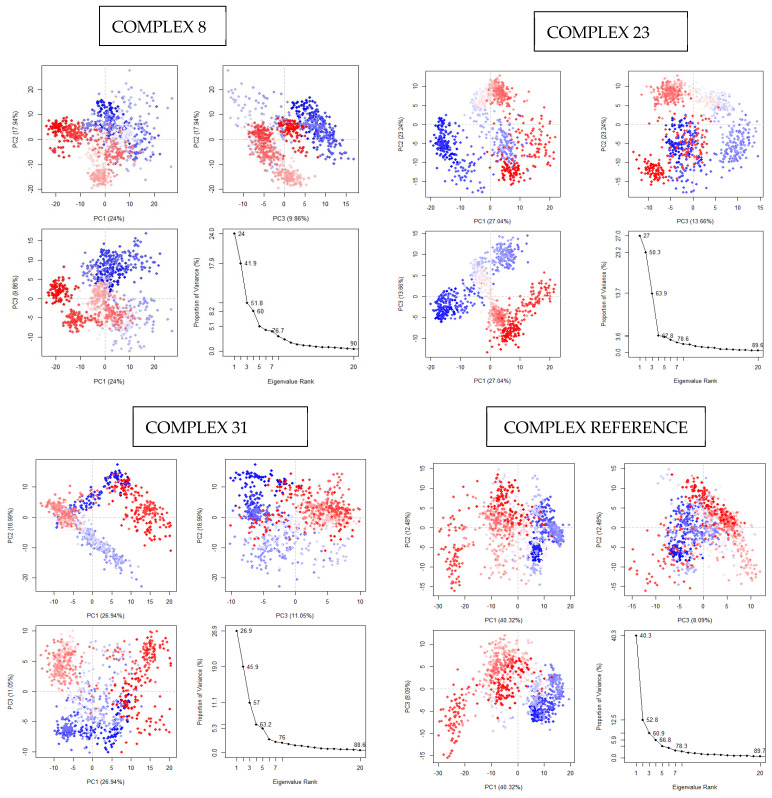
PCA (principal component analysis) profiles for Compounds 8, 23, and 31 and bromosporine complexes.

The statistical technique Principal Component Analysis (PCA) reduces the dimensionality of data. In this instance, the ligand-induced significant changes in the backbone C atoms through its application. This technique distinguishes between the residual and essential protein motions. The critical motions, usually the largest-scale movements, frequently include biologically important movements like opening, shutting, and flexing. The remaining motions represent minor, less significant local changes [50]. Figure 7 shows that the first three PCs in the simulations for TcBDF2, TcBDF2 Compound 8, TcBDF2 Compound 23, and TcBDF2 Compound 31 accounted for 24%, 27.04%, and 26.94% of the total number of variances in the generated data. These numbers illustrate the range of simulated conformations. The systems bound to Compounds 8, 23, and 31 exhibited slightly wider ranges. These ligands cause stronger structural motions than the reference ligand (bromosporine). Additionally, we observed a total variance of 90% for the first principal component in Complex 8. Likewise, the variation was 89.6% for Complex 23 and 88.6% for Complex 31. The results prove that Complex 8 enhanced the motion of the protein’s Cα-atoms. Accordingly, this data implied that ligand interaction modifies TcBDF2′s conformation.

#### 3.4.7. Dynamic Cross Correlation Matrix (DCCM)

**Figure 8:**
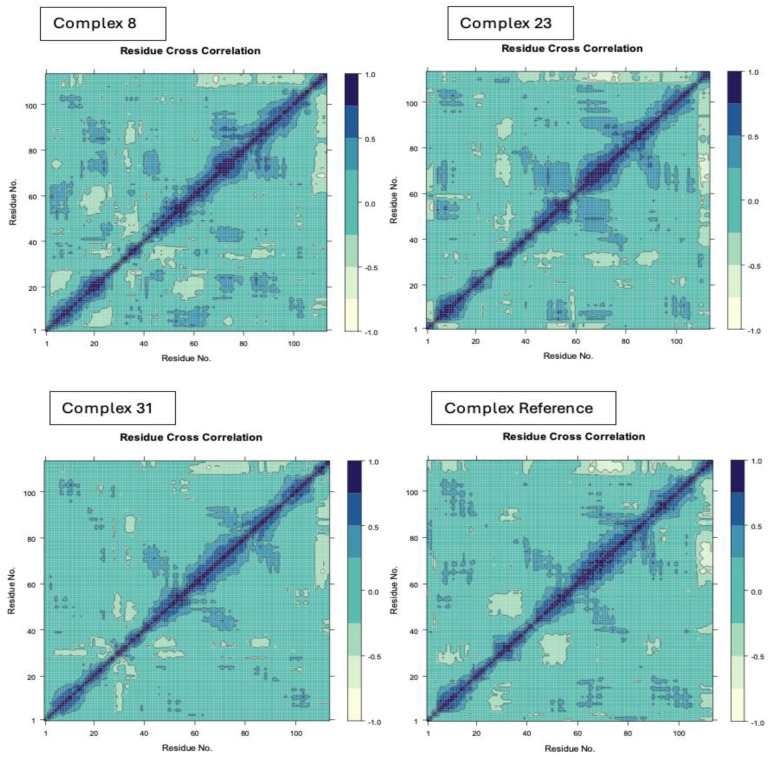
DCCM (dynamic cross-correlation matrix) profiles for Compounds 8, 23, and 31 and bromosporine complexes.

A DCCM plot was made to examine the correlated movement of structural domains to reach a stable conformational state of the complex after binding to the chosen compounds (Figure 8). The matrix plot’s distinct color patterns represent varying correlation levels [51]. The positively correlated motions are depicted in dark blue, while negatively anti-correlated motions are shown in white, and mixed correlations are illustrated in cyan. Complex eight reduced anti-correlated movements while preserving moderate correlated motions in TcBDF2, indicating stable and correlated residue dynamics. Next, the compound lowered correlated motions and created widespread anti-correlated motions. This made TcBDF2 more flexible and less stable structurally. Subsequently, Complex 31 maintained correlated motions while producing localized anti-correlated motions, indicating moderate stability and flexibility in TcBDF2. The DCCM analysis showed that complex 8 induced a robust open structure ensemble in the protein, which was ascribed to the flexibilities of various residues in this state, despite both systems (Complexes 23 and 31) exhibiting both open and closed conformations in comparison with the bromosporine.

Chandrananimycin A, which is also called N-(9-hydroxy-3-oxophenoxazin-2-yl) acetamide, is a new bioactive compound that was found in Actinomadura sp.a. sp. This compound exhibited broad-spectrum biological properties, including antibacterial, antiviral and anticancer activities. Maskey et al.’s 2003 study demonstrated the activity of Chandrananimycins against human colon carcinoma CCL HT29; melanoma MEXF 514L; lung carcinoma LXFA 526L and LXFL 529L; breast carcinoma CNCL SF268, LCL H460, and MACL MCF-7; prostate cancer PRCL PC3M; and renal cancer RXF 631L cell lines, with IC70 values as low as 1.4 g/mL [52].

Venezueline A, also known as N-[7-[(3-acetamido-4-hydroxyphenyl)methyl]-8-(hydroxymethyl)-3-oxophenoxazin-2-yl]acetamide was isolated from Streptomyces venezuelae. A study by Ren et al. (2013) reported the antitumor effect of phenoxazine-type alkaloids, including the five new venezuelines (A-E) related to the upregulation of the gene target Nur77 [53].

According to research by Ebada and colleagues (2015), Dispacamide E and other bromopyrrole alkaloids exhibit strong and specific activities against one or more kinases, making them promising candidates for the creation of novel treatments for diabetes, cancer, Alzheimer’s disease, and malaria [54].

We looked at the results of chandrananimycin A, venezueline A, and dispacamide and compared them to BZN and NFX, which are the current standard treatments for Chagas disease. Despite the efficacy of BZN and NFX in reducing parasitemia during the acute phase, their use has resulted in significant adverse effects, including gastrointestinal and neurological toxicity, as well as reduced effectiveness in chronic cases. On the other hand, the compounds under study demonstrate high binding affinities to key *T. cruzi* targets and display favorable pharmacokinetic properties. Further experiments could validate the potential advantages of chandrananimycin A, venezueline A, and dispacamide in terms of safety and potency. Moreover, chandrananimycin A shares similar structural properties with quinone-based compounds, which have been proven to be broad-spectrum antiparasitic and antibacterial. Venezueline A is structurally similar to macrolides, known for their antiparasitic action and immunomodulatory effects, potentially leading to new therapeutic applications. Dispacamide, which has well-defined anti-inflammatory effects, could also tackle the inflammatory damage associated with chronic CD through a mechanism of action different from those of BZN and NFX.

Researchers have discovered that marine-derived compounds, through multiple mechanisms beyond binding to *T. cruzi* proteins, exert a wide range of therapeutic effects, including antiparasitic activity. These mechanisms encompass the inhibition of the major cysteine proteases, such as cruzipain, the disruption of the trypanothione redox metabolism, and interference with parasite replication or energy production pathways. Furthermore, specific marine-derived bioactive metabolites may destabilize parasite membrane integrity or modulate host-parasite interactions, reducing parasite survival.

Using standardized culture systems, we can perform in vitro tests with marine-derived compounds on *T. cruzi* at different life stages (epimastigotes, trypomastigotes, and amastigotes). High-throughput screening technologies allow the rapid characterization of compound libraries and select promising candidates with activity in a specific stage. The cell-fraction-based cytotoxicity assays, along with emerging techniques like cellular imaging and metabolomic profiling, hold valuable potential to explore the selectivity of these compounds toward *T. cruzi* compared to mammalian hosts. Further in vivo validation could be performed using murine models for acute and chronic CD. In acute infection models, the compounds are assessed for their capacity to decrease parasitemia and control inflammation in cardiac tissues. Parasite load can be measured in chronic models by quantitative PCR and histopathological examination of affected organs. In addition, recent advances in molecular imaging techniques have allowed monitoring of compound biodistribution and interactions with target tissues in vivo, whereas biomarkers are used to track systemic toxicity. These approaches provide extensive pharmacokinetic, efficacy, and safety data on marine compounds for therapeutic development. Similar studies done on related microorganisms like Plasmodium falciparum and Leishmania species have confirmed that metabolites from marine sources have broad antiparasitic activity. This highlights their potential usefulness as new therapeutic candidates [55,56].

The previous suggested studies will reinforce our result from the virtual screening of marine natural products, and the TcBDF2 inhibitory potential of compounds Chandrananimycin A, venezueline A, and dispacamide will be validated. This study concluded that the three based-marine compounds exhibited exceptional interaction with the active site of the TcBDF2. MD simulations and post-MD analyses also showed that there are possible inhibitor candidates that keep the molecular docking complexes stable. Additionally, the selected compounds showed favorable pharmacokinetic characteristics. The findings suggest that each of these compounds might serve as a CD therapeutic candidate and may also pave the path for the future development of efficient treatments and CD prevention strategies. The computational nature of this study necessitates further validation through in vitro and in vivo tests.

Future research should conduct rigorous validation of the compounds identified in this study as therapeutic agents, both in vitro and in vivo. We should improve pharmacokinetics and pharmacodynamics to maximize efficacy while minimizing potential toxicity. Investigating structurally related natural analogs and employing advanced methodologies in drug development may further aid in identifying promising therapeutic candidates. Interdisciplinary collaboration will be crucial in overcoming challenges and facilitating the development of effective treatments for neglected tropical diseases.

## 4. Conclusions

Chagas disease poses a major public health challenge, particularly in resource-constrained regions. Existing treatment options, while available, suffer from significant limitations and adverse effects. Leveraging computer-aided drug design (CADD) approaches and exploring natural compounds as potential drug sources offer promising avenues for developing more effective and safer therapies. Such advancements could enhance patient outcomes and reduce the global burden of this neglected tropical disease. In this study, we employed advanced computational techniques to virtually screen over 32,000 marine-derived compounds from the CMNPD database, identifying candidates with potential therapeutic value. These helped us find three compounds—chandrananimycin A (Complex 8), venezueline A (Complex 23), and dispacamide (Complex 31)—that might be useful as medicines to treat Chagas disease. The proposed hit compounds represent a promising foundation for the development of new trypanocidal agents derived from natural products. Importantly, these candidates adhere to Lipinski’s Rule of Five, with none violating more than one criterion, and demonstrate favorable toxicity and pharmacokinetic profiles, highlighting their potential for further drug development. We conducted our research exclusively using computational approaches. Given the predictive limitations inherent in computational biology methods used in drug discovery, we must perform future wet lab experiments to validate the findings of our study.

## Figures and Tables

**Table 1:** The drug-likeness properties and binding affinities in kcal/mol of the 31 best ligand.

Molecule	Binding Affinity in kcal/mol	MW ^1^	HBA ^2^	HBD ^3^	iLOGP	Lipinski # Violations	PAINS # Alerts
1	−10.2	797.07	10	8	7.98	2	0
2	−10.1	746.49	6	9	2.78	3	0
3	−9.8	857.86	17	13	1.99	3	1
4	−9.4	594.52	13	7	2.2	3	1
5	−9.3	789.74	13	13	3.13	3	1
6	−9.3	254.29	2	2	1.87	0	1
7	−9.1	844.75	10	8	3.69	3	0
8	−9.1	270.24	5	2	2.02	0	0
9	−9.1	458.48	4	8	−0.14	2	0
10	−9	802.99	16	6	4.82	3	0
11	−9	610.52	14	8	0.92	3	1
12	−9	784.44	6	10	−2.01	3	0
13	−9	726.72	15	6	3.62	3	0
14	−9	694.74	10	13	0.28	3	0
15	−8.9	741.41	7	9	−0.2	3	0
16	−8.9	636.3	5	9	0.39	3	0
17	−8.9	333.18	2	2	2.31	0	1
18	−8.9	610.52	14	8	2.22	3	1
19	−8.9	898.2	8	10	1.79	3	0
20	−8.9	899.21	7	9	1.98	3	0
21	−8.8	900.21	6	9	0.61	3	0
22	−8.8	599.59	11	6	1.15	3	0
23	−8.8	447.44	7	4	2.64	0	0
24	−8.7	199.23	1	2	−2.01	0	0
25	−8.7	450.57	5	2	4.49	0	0
26	−8.7	969.48	7	3	7.6	2	0
27	−8.7	808.12	5	8	0.35	3	0
28	−8.7	928.34	10	4	5.44	2	0
29	−8.6	780.11	4	8	2.07	3	0
30	−8.6	661.51	8	10	1.23	3	0
31	−8.6	405.05	3	4	1.05	0	0

^1^ Molecular weight, ^2^ Hydrogen bond acceptors, ^3^ Hydrogen bond donors.

**Table 2:** ADMET profiles of short-listed compounds.

Molecule	Intestinal Absorption Human (% Absorbed)	BBB Permeability (log BB)	CYP2D6 Inhibitor	CYP3A4 Inhibitor	Total Clearance (log mL/min/kg)	AMES Toxicity
8	88.159	−0.141	No	No	0.334	No
23	80.906	−1.055	No	No	−0.067	No
24	94.67	0.435	Yes	No	0.918	Yes
25	92.268	−0.628	No	Yes	1.485	Yes
31	70.422	−1.072	No	No	0.892	No
BZN	75.5	−0.863	No	No	0.519	Yes
NFX	83.905	−0.953	No	No	0.718	Yes

**Table 3:** Interaction for the selected hit molecules within the binding pocket of TcBDF2.

Complex ID	CMNPD ID	ΔG kcal/mol	Involved Residues	Interactions
8	CMNPD13212	−9.1	Met 30, Tyr 43 Ala 82 Trp 92 Ile 35, Leu 40	Conventional hydrogen bond Carbon hydrogen bond Pi–pi stacked interaction Pi-Alkyl interaction
23	CMNPD23228	−8.8	His 33, Phe 31, Val 51, Met 78, Asp 52, Asn 81, Asn 86, Tyr 85, Asp 42, Thr 90 Met 30, Tyr 43, Ala 87 Ala 82 Trp 82 Ile 35, Leu 40	van der Waal’s interaction Conventional hydrogen bond Carbon hydrogen bond Pi–pi stacked interaction Pi–alkyl interaction
31	CMNPD8246	−8.6	Pro 34, Asp 52, Tyr 85 Val 51, His 33, Met 30, Asn 86 Met 78 Phe 31, Tyr 43, Trp 92 Ile 35, Ala 82, Leu 40	van der Waal’s interaction Conventional hydrogen bond Carbon hydrogen bond Pi–pi stacked interaction Pi–alkyl interaction
Reference	Bromosporine	−7.7	Glu 91, Lys 29, Tyr 85, Tyr 43 Leu 28, Trp 92, Asn 86 Met 30 Trp 92 Leu 40, Ala 82, ILe 35, Phe 31	van der Waal’s interaction Conventional hydrogen bond Pi–sigma interaction Pi-Sulfur interaction Pi–alkyl interaction

## Data Availability

All the data generated during the study are included in the manuscript. No additional data are available to share as research data.
